# Headliners: Breast Cancer: GATA-3 Maintains Differentiation of Mammary Ductal Cells

**Published:** 2007-03

**Authors:** Jerry Phelps

Kouros-Mehr H, Slorach EM, Sternlicht MD, Werb Z. 2006. GATA-3 maintains the differentiation of the luminal cell fate in the mammary gland. Cell 127:1041–1055.

*GATA-3* is one of a family of genes responsible for driving the processes that turn undifferentiated stem cells into specialized mature cells. Now NIEHS grantee Zena Werb of the University of California, San Francisco, and colleagues have determined that the GATA-3 protein is also required for the maintenance of differentiation in ductal cells of the mammary gland. This new finding suggests that *GATA-3* may play a key role in the development of breast cancer.

Mammary ductal cells, also known as luminal cells, line the mammary ducts that carry milk during lactation. Although not much is known about the differentiation of luminal cells, they are implicated as a primary site in the mammary gland for cancers to form. Cancer researchers know that breast tumors with high GATA-3 expression have a good prognosis. These cancers tend to be well-differentiated, and the cells maintain many characteristics of normal mammary cells, including high numbers of estrogen receptors. However, cancers with low expression of the protein tend to be diffuse and poorly differentiated, and lead to poor prognosis for the patient.

Upon devising a microarray strategy to identify novel regulators of mammary development, the investigators observed the mammary epithelium *in vivo* of laboratory mice genetically altered to lack *GATA-3*. They found that mature cells reverted to the less specialized, undifferentiated state, which is characteristic of aggressive cancer cells. The research team also found GATA-3 in all mammary duct luminal cells in normal mice at puberty and into adulthood.

The results suggest that the loss of functioning genes and the subsequent failure to maintain the mature state of the cells is what leads to the loss of differentiation and uncontrollable proliferation during cancer progression. Prior to this finding, it was unclear that maintaining differentiation of mammary cells was an active process and that the GATA-3 protein was responsible for that maintenance. The team is now studying how GATA-3 controls cell fate and its role in breast cancer. This research could also have implications in other cancer types.

## Figures and Tables

**Figure:**
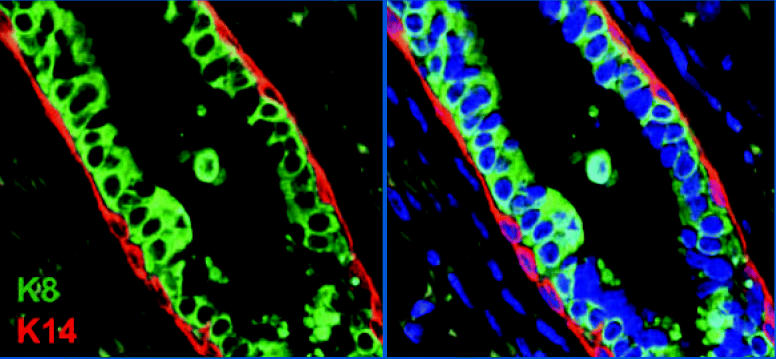
Loss of *GATA-3* leads to expansion of undifferentiated luminal cell population

